# Self-reported generalised pruritus among community-dwelling older adults in Malaysia

**DOI:** 10.1186/s12877-020-01610-y

**Published:** 2020-06-24

**Authors:** Shin Shen Yong, Zhenli Kwan, Chin Chwen Ch’ng, Adrian Sze Wai Yong, Leng Leng Tan, Winn Hui Han, Shahrul Bahyah Kamaruzzaman, Ai-Vyrn Chin, Maw Pin Tan

**Affiliations:** 1 Division of Dermatology, Department of Medicine, Faculty of Medicine, University of Malaya, 50603 Kuala Lumpur, Malaysia; 2 Division of Geriatric Medicine, Department of Medicine, Faculty of Medicine, University of Malaya, Kuala Lumpur, Malaysia

**Keywords:** Comorbidity, Dermatology, Generalised pruritus, Geriatrics medicine, Sleep quality

## Abstract

**Background:**

The ageing skin is more susceptible to pruritic dermatoses, which are associated with adverse psychosocial effects and reduced quality of life among older adults. This cross-sectional study aimed to identify the burden of pruritus and factors associated with its presence and severity among older adults recruited to the Malaysian Elders Longitudinal Research study.

**Methods:**

Seven hundred seventy individuals aged 55 years (lower age limit) and above were interviewed as to whether they experienced pruritus during the preceding week and the locations involved if present. The association between generalised pruritus, sleep quality, and major systemic risk factors were explored.

**Results:**

5.97% of respondents reported generalised pruritus. Generalised pruritus was associated with poorer sleep quality, with Pittsburgh Sleep Quality Index score above 6. Mean haemoglobin level was lower in subjects with generalised pruritus (13.14 g/dL) but there was no significant difference in the frequency of generalised pruritus and severe generalized pruritus between subjects with clinically defined anaemia. Also, there were no significant associations between other major systemic risk factors and generalised pruritus in this population-based study. There was no association between generalised pruritus with depression, anxiety or stress.

**Conclusion:**

The negative effect of pruritus on sleep quality suggests a possible deleterious effect of pruritus on health and quality of life. Further prospective research on the longer-term effects of pruritus on health status is now warranted.

(222 words)

## Background

In 2015, there were 2.8 million individuals aged 60 years and over in Malaysia, which comprises over 9% of its total population of 31 million [1]. Therefore, Malaysia is expected to achieve the status of an ageing nation status by 2035 when senior citizens make up 15% or 5.6 million of its population [2]. Older patients represent a unique population group with regards to skin disorders. Among individuals within the older age group, symptoms of pruritus may represent systemic disease and require a comprehensive clinical approach. Furthermore, the presence of chronic pruritus is often associated with long-term discomfort adversely affects the quality of life and psychological well-being [3, 4].

The International Forum for the Study of Itch (IFSI) classification categorises itchiness into three groups, namely: pruritus on the diseased skin (group I), non-diseased skin (group II) and secondary lesions due to pruritus (group III). Underlying diseases are classified into dermatological, systemic, neurological, psychogenic, mixed, and other causes [5]. Skin ageing increases the susceptibility to pruritic dermatoses [6]. The reduction in skin cell turnover and barrier function together with lifestyle factors such as sleep quality, stress, and diet are the factors associated with skin ageing [7].

Pruritus, or itch, is a common symptom or presentation in many systemic, dermatological, or psychological disorder. Any tissue damage or inflammation can lead to localised or generalised pruritus and pruritus can be defined as the sensation that can be relieved by scratching of the skin [8]. Published data on the prevalence of pruritus and factors associated with itch among older populations remain limited. Much of previous data focused on institutionalised residents and patients attending geriatric outpatient clinics rather than community-dwelling adults [9–11]. An understanding of the magnitude of the problem, associated risk factors, and possible impact on the general population will aid in resource planning as well as inform clinical practice. We, therefore, determined the burden of generalised pruritus as well as factors associated with its presence and severity among the community-dwelling older population.

## Methods

This study utilized data from the first and second waves of the Malaysian Elders Longitudinal (MELoR) study. The measurement of pruritus, psychological status, sleep quality and self-rated health are described below.

### Sample population

The MELoR study is a cohort study comprising of community-dwelling older adults aged 55 years or over at the time of recruitment between 2012 and 2015. Participants, in the first wave study, were selected through simple random sampling stratified by age and ethnicity from the electoral rolls of the parliamentary constituencies of Petaling Jaya North, Petaling Jaya South, and Pantai Valley. This study recruited 1623 participants from the community. Two hundred seventy-four with incomplete data were excluded and the remaining 1349 participants were included for follow-up. However, 45 died and 534 were unable to follow up due to insufficient resources, therefore only 770 completed the follow-up assessments in the second wave study. The study methods have been described in detail elsewhere [12]. This study was approved by the University of Malaya Medical Centre Medical Ethics Committee (Ref: 925.4) and complied with the Helsinki Declaration of 1975, revised in 1983. Written informed consent was obtained from all study participants prior to their inclusion.

### Data collection

First wave data were collected through a detailed home-based computer-aided questionnaire interview followed by a hospital visit for clinical assessments. Information on basic demographics, socioeconomic status, living environment, health, psychology, quality of life, function, social participation, lifestyle and health beliefs were obtained at baseline. Eligible participants were then interviewed again in the second wave between 2015 and 2016. Changes in health, psychological, functional, social, lifestyle, and quality of life outcomes were measured using a computerised questionnaire survey in the second wave, as in the first wave. Second wave data were obtained entirely from a home-based questionnaire survey administered by the trained researchers.

Changes in health, psychological, functional, social, lifestyle, and quality of life outcomes were measured using a computerised questionnaire survey. Psychological symptoms were assessed with the 21-item Depression, Anxiety and Stress Scale (DASS-21), perceived health status using self-rated health, and sleep quality suing the Pittsburgh Sleep Quality Index (PSQI). For the purpose of this study, medical history from both the first wave and second wave were merged to establish prevalence of each condition at the second wave. Only DASS-21 results from the second wave was included in this analysis.

The DASS-21 consists of seven items each for each domain of depression, anxiety and stress rated on a four-point Likert scale, with 0 indicating “did not apply to me at all”, and 3 indicating “applied to me all the time”. The sum of scores are calculated for each domain, and the total score is then multiplied by two. The maximum score for each domain is therefore 42, with 0 indicated absence of any symptoms and 42 indicating extremely severe symptom. Symptoms of depression, anxiety, or stress symptoms were considered present for those with score of 10 or above, 8 and above and 15 and above for the respective domains [13].

The Pittsburgh Sleep Quality Index was used to assess the quality and patterns of sleep by evaluating seven components of sleep. It contains 19 self-rated questions and 5 questions rated by the bed partner or roommate (if available). Only self-rated questions are included in the scoring. The 19 self-rated items are combined to form seven “component” scores, each of which has a range of 0–3 points. In all cases, a score of “0” indicates no difficulty, while a score of “3” indicates severe difficulty. The component scores were summed to produce a global score ranging from 0 to 21. A score of 5 or higher indicated poor sleep quality [14].

Self-rated health (SRH) was assessed using the two questions, “Would you say your health is……?” and “In general, compared to other people your age, would you say your health is…….? The possible responses were: “poor”, “fair”, “good”, “very good” and “excellent”. Response for each question was then dichotomized to “poor or fair” and “good, very good or excellent”.

### Pruritus

Participants were asked at the second wave whether they had experienced pruritus in the preceding week. Those who responded ‘yes’ to the presence of pruritus in the preceding week were then asked whether the pruritus was localised or generalised. They were also asked to rate the severity of pruritus on a scale on 0–10 using a numerical rating scale (NRS) which represents a row of 10 rectangles with increasingly heaving shading starting from no shading for 0–1 and black solid shading from 9 to 10. Numerical numbers from 0 to 10 were also included beneath the margin between each rectangular box, and at each end. The NRS is a unidimensional scale which has similarly been validated for measuring the severity of pain, where 0 represents “having no symptoms” while ten represents “having the worst imaginable symptoms.” For NRS, a score of 0 to 2 represents no or mild pruritus, a score of 3–6 represents moderate pruritus, while a score of 7 to 8 and 9 to 10 represents severe and very severe pruritus, respectively [15]. For chronic pruritus, the NRS, visual analogue scale (VAS) and verbal rating scale (VRS) have shown high reliability and concurrent validity (r > 0.8; p < 0.01) [16].

### Statistical analysis

Data were analysed using the SPSS version 21.0 (IBM Corporation). Essential characteristics obtained at wave one was then compared against those with and without pruritus. For parametric data, the independent t-test was used to compare means while the Mann-Whitney U test was used to compare medians for non-parametric data. The Pearson chi-squared test or Fisher’s exact test where necessary were used to determine possible factors associated with generalised pruritus. All tests were two-tailed with a significance level of p < 0.05. Potential confounders were adjusted for by using multivariate logistic regression with stepwise backward method. The fit of the logistic models was assessed on the basis of the Hosmer-Lemeshow test and p < 0.05 (two tailed) was considered as statistically significant.

## Results

### Generalised pruritus

**Fig. 1:**
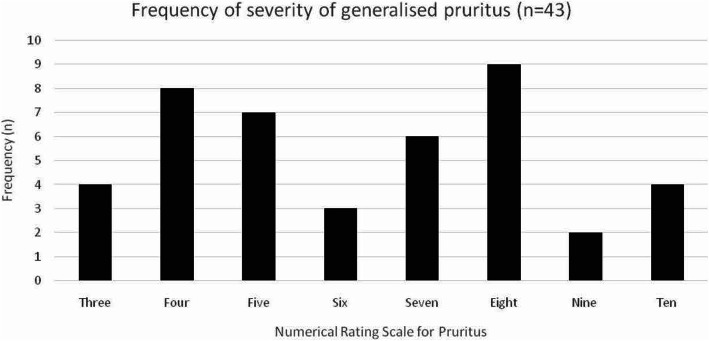
Severity grading of generalised pruritus. The frequency and severity of generalised pruritus among 43 patients who reported generalised pruritus

Data on the presence or absence of pruritus were available for 770 subjects interviewed during the second wave. The demographics of the study population are as shown (Table 1). The median age was 69.86 years (interquartile range 64.70 years and 75.20 years). For subjects reporting generalised pruritus, the median age was 70.81 years while for those without generalised pruritus, the median age was 69.83 years. This difference was not statistically significant using the Mann-Whitney U test (p = 0.880). Forty-six subjects (5.97%) reported generalised pruritus, with 45.7% (n = 21) reporting either severe or very severe pruritus (Fig. 1).

**Table 1:** Demographics of the study population

Demographic	Frequency, n (%)
**Gender**	
Male	331 (43.0)
Female	439 (57.0)
**Marital status**	
Married	545 (70.8)
Widowed	152 (19.7)
Single/never married	50 (6.5)
Divorced/separated	20 (2.6)
Other relationship (i.e. life partner)	1 (0.1)
Unknown	2 (0.3)
**Education**	
Secondary	327 (42.5)
College/university	225 (29.2)
Primary	150 (19.5)
Certificate/skill (post-secondary)	42 (5.5)
No formal education	22 (2.9)
Unknown	4 (0.5)

### Risk factors for generalised pruritus

The mean haemoglobin for subjects who reported generalised pruritus was 13.14 g/dL (standard deviation, SD = 1.217) compared to 13.71 g/dL (SD = 1.487) for those without generalised pruritus (t = − 2.966, mean difference − 5.75, p = 0.005). However, there was no significant difference in the frequency of genaralized pruritus and severe generalized pruritus between subjects with clinically defined anemia and those without (p = 0.275 and p = 0.507, respectively). We did not find any association between generalised pruritus and other possible risk factors such as chronic kidney disease, thyroid disorders and liver disease (Table 2). The association between pruritus and sleep remained significant even after adjustment for haemoglobin. (adjusted OR 0.516, p = 0.049, 95% CI 0.267–0.997). (Table 3).

**Table 2:** Associations between generalised pruritus and selected systemic factors

Factors	Generalised pruritus		Total	χ^2^	p-value	Unadjusted Odds Ratio (OR)	95% Confidence interval	
	Yes	No					Lower	Upper
**Age**	–	–	–	–	0.880	0.997	0.961	1.035
**Gender**								
Male	14 (30.43%)	317 (43.78%)	331	3.145	0.076	0.562	0.295	1.071
Female	32 (69.57%)	407 (56.22%)	439					
**Heart disease**							1.046	1.084
Yes	0 (0.0%)	15 (2.07%)	15	0.972	1.000	1.065		
No	46 (100%)	709 (97.93%)	755					
**Hypertension**								
Yes	28 (60.87%)	465 (64.23%)	493	0.212	0.646	0.866	0.470	1.597
No	18 (39.13%)	259 (35.77%)	277					
**Dyslipidaemia**								
Yes	34 (73.91%)	539 (74.45%)	573	0.006	0.936	0.972	0.493	1.918
No	12 (26.09%)	185 (25.55%)	197					
**Malignancy**								
Yes	3 (6.52%)	28 (3.87%)	31	0.789	0.423	1.734	0.507	5.932
No	43 (93.48%)	696 (96.13%)	739					
**Parkinson**								
Yes	0 (0.0%)	4 (0 .55%)	4	0.255	1.000	1.064	1.045	1.083
No	46 (100%)	720 (99.45%)	766					
**Depression**								
Yes	1 (2.17%)	17 (2.35%)	18	0.006	1.000	0.924	0.120	7.102
No	45 (97.83%)	707 (97.65%)	752					
**Anxiety**								
Yes	0 (0.0%)	15 (2.07%)	15	0.972	1.000	1.065	1.046	1.084
No	46 (100.0%)	709 (97.93%)	755					
**Diabetes mellitus**								
Yes	26 (56.52%)	330 (45.58%)	356	2.083	0.149	1.522	0.851	2.831
No	20 (43.48%)	394 (54.42%)	414					
**Chronic kidney disease**								
Yes	5 (10.87%)	83 (12.15%)	88	0.015	0.902	0.942	0.362	2.450
No	41 (89.13%)	641 (87.85%)	682					
**Thyroid disease**	2 (4.35%)	39 (5.39%)	41	0.093	1.000	0.798	0.187	3.415
Yes	44 (95.65%)	685 (94.61%)	729					
No								
**Liver disease**								
Yes	1 (2.17%)	12 (1.66%)	13	0.070	0.554	1.319	0.168	10.367
No	45 (97.83%)	712 (98.34%)	757					
**Gout**								
Yes	6 (13.04%)	52 (7.18%)	58	2.133	0.148	1.938	0.786	4.783
No	40 (86.96%)	672 (92.82%)	712					
**Hyperuricemia**								
Yes	7 (15.22%)	103 (14.23%)	110	0.035	0.852	1.082	0.471	2.484
No	39 (84.78%)	621 (85.77%)	660					
**Alcohol intake**								
Yes	15 (32.61%)	239 (33.01%)	254	0.003	0.955	0.982	0.520	1.854
No	31 (67.39%)	485 (66.99%)	516					

*p < 0.05

Chronic kidney disease (CKD) = CKD stage 3 and above based on glomerular filtration rate (GFR) or self-reported CKD

**Table 3:** Association between pruritus and sleep remained significant even after adjustment for haemoglobin

Predictors	B	S.E.	Wald	df	p-value	Adjusted odds ratio	95% CI	
							Lower	Upper
**Haemoglobin**	−0.027	0.011	6.246	1	0.012	0.974	0.953	0.994
**Sleep Quality**	−0.661	0.336	3.880	1	0.049	0.516	0.267	0.997
**Constant**	1.774	1.490	1.417	1	0.234	5.894		

We categorised respondents according to their ages. For those aged between 55 and less than 65 years old, 6.5% (n = 13) reported generalised pruritus while for those aged between 65 to less than 75 years old, the corresponding figure was 5.9% (n = 22). Similarly, for those aged between 75 to less than 85 years old, 6.4% (n = 11) had generalised pruritus. None of the respondents aged 85 years and above reported similar complaints. Further analysis did not reveal any statistically significant differences between the different age categories in terms of frequency or severity of generalised pruritus among the study respondents.

### Impact of generalised pruritus

Poor Pittsburgh Sleep Quality Index (PSQI) was associated with generalised pruritus (p = 0.04). However, generalised pruritus was neither associated with general health nor psychological comorbidities such as depression, anxiety and stress (Table 4).

**Table 4:** Associations between generalised pruritus and self-rated health, psychological status and sleep quality according to the Pittsburgh Sleep Quality Index (PSQI)

	SRH1 (n = 747)		Total	χ^2^or Fisher’s exact test	p-value
	Poor orW fair	Good, very good or excellent			
Generalised pruritus:					
Yes	20 (43.5%)	26 (56.5%)	46	0.117	0.732
No	323 (46.1%)	378 (53.9%)	701		
	SRH2 (n = 738)				
	Poor or fair	Good, very good or excellent			
Generalised pruritus:					
Yes	15 (32.6%)	31 (67.4%)	46	0.474	0.491
No	193 (27.9%)	499 (72.1%)	692		
	Depression (n = 749)				
	Yes	No			
Generalised pruritus:					
Yes	7 (15.6%)	38 (84.4%)	45	1.732	0.188
No	67 (9.5%)	637 (90.5%)	704		
	Stress (n = 751)				
	Yes	No			
Generalised pruritus:					
Yes	1 (2.2%)	44 (97.8%)	45	0.537	0.463
No	32 (4.5%)	674 (95.5%)	706		
	Anxiety (n = 749)				
	Yes	No			
Generalised pruritus:					
Yes	6 (13.3%)	39 (86.7%)	45	0.017	0.897
No	99 (14.0%)	607 (86.0%)	706		
	Sleep Quality(n = 756)				
	Poor	Good			
Generalised pruritus:					
Yes	31 (67.4%)	15 (32.6%)	46	4.348	0.047*
No	366 (51.5%)	344 (48.5%)	710		

*SRH* self-rated health, *SRH1* general SRH, *SRH2* SRH compared to people of similar age. *p < 0.01

## Discussion

Six percent of our older community dwelling population aged 55 years and over reported the presence of generalised pruritus over the previous week, of which almost half described severe or very severe pruritus. The presence of pruritus was associated with lower haemoglobin levels and reduced sleep in our population.

While increasing age is associated with increased risk of multiple morbidities such as liver and renal disease with are in turn associated with itch, the presence of itch in our general older population was not found to be associated with any specific medical condition. This implies that pruritus in our older population may be primarily attributed to specific dermatoses, which may necessitate evaluation by trained physicians in order to institute appropriate treatment measures to ameliorate the symptoms. In this study, despite the significant difference in the mean haemoglobin between respondents who reported generalised pruritus and those who did not, the difference was too small to draw any clear conclusions in terms of clinical significance. This concurred with the study done by Yonova et al., where pruritus was not common among elderly people with anaemia but there is a possible role of iron deficiency [17]. As iron is implicated in the key functions of many enzymes, its deficiency may lead to alteration in enzymatic function and metabolic function with subsequent itching [18]. Cyanocobalamin levels have also been reported to be lower in patients with generalised pruritus compared to controls [19]. Hence, further research can be conducted to investigate the role iron deficiency in generalised pruritus in the elderly population. However, we were unable to proceed to test subjects for nutritional deficiencies due to funding issues and this may be an area for potential future work.

Older individuals with pruritus in our study population were not more likely to have poorer self-rated health, depression, anxiety and stress [19]. Our findings conflicted with that of other previous studies which evaluated pruritus in dermatology clinics which reported the presence of negative psychological sequelae from pruritus associated with dermatological conditions, such as prurigo nodularis, atopic dermatitis, and pruritus of unknown origin [4]. In addition, pruritus has often been cited as a distressing symptom affecting quality of life among patients with end-stage renal disease [20]. We can therefore surmise that although patients with specific dermatological conditions or chronic medical conditions associated with pruritus may experience psychological effects, this does not apply in the general older population who report the presence of generalised pruritus.

Pruritus, however, is associated with impaired sleep quality in our study which is consistent with the findings of previous studies [3]. In the absence of a direct relationship between itch and negative psychological symptoms, the negative effects of reduced sleep resulting from pruritus remains undefined in this study. Reduced sleep has been associated with reduced physical activity and reduced likelihood to go outdoors in a previous study [21]. The lack of sunlight exposure and physical exercise then further exacerbates the reduction in sleep quality. This could be due to the increment of melatonin level from adequate sun exposure and shorter sleep onset latency with regular exercise [22]. Poor sleep quality in those with pruritus may also be drug-induced. Corticosteroids [23] and first generation H1 antagonist [24] can cause hyperactivity, insomnia, and anxiety due to the blockade of acetylcholine receptors [25]. The act of scratching can also lead to lightening of sleep and a longer bout of scratching is associated with higher likelihood of subsequent arousal [26].

Our findings are limited by the cross-sectional relationship between pruritus and other associated factors. As pruritus was not recorded in the first wave of MELoR, we were unable to establish new incidence or a prospective relationship between itch and clinical outcomes. Due to resource constraints, it was not possible to assess the individuals with itch clinically to establish the underlying causes of their pruritus, and only half of the initial cohort were evaluated in the second wave due to an abrupt withdrawal of funding. Furthermore, the small number of participants with itch may also limit our ability to detect associated psychological effects. A larger study would be required to further determine the burden of pruritus in the general older population, but our findings will be vital to inform the power calculations for such a study.

## Conclusion

Generalised pruritus is common among community-dwelling older adults aged 55 years and over and the presence of pruritus is leads to poorer sleep quality. Those with pruritus also had lower haemoglobin. Self-reported kidney and liver diseases, however, were not significantly associated with general pruritus. Future larger studies will now need to establish the likely aetiology of pruritus in the general older population to accurately determine the burden of this common condition.

## Data Availability

The datasets used and/or analysed during the current MeLOR study are available from the corresponding author on reasonable request.

## Abbreviations

IFSI: The International Forum for the Study of Itch
MeLOR: Malaysian Elders Longitudinal Study
DASS-21: Depression, Anxiety and Stress Scale-21
PSQI: Pittsburgh Sleep Quality Index
SRH: Self-rated health
NRS: Numerical rating scale
VAS: Visual analogue scale
VRS: Verbal rating scale
